# Metacognition and evidence analysis instruction: an educational framework and practical experience

**DOI:** 10.1186/s13643-015-0101-8

**Published:** 2015-08-21

**Authors:** J. Scott Parrott, Matthew L. Rubinstein

**Affiliations:** Department of Interdisciplinary Studies, Rutgers, The State University of New Jersey, 65 Bergen Street, Room 353A, Newark, NJ 07107 USA

**Keywords:** Evidence analysis, Instruction, Critical thinking, Metacognition

## Abstract

The role of metacognitive skills in the evidence analysis process has received little attention in the research literature. While the steps of the evidence analysis process are well defined, the role of higher-level cognitive operations (metacognitive strategies) in integrating the steps of the process is not well understood. In part, this is because it is not clear where and how metacognition is implicated in the evidence analysis process nor how these skills might be taught.

The purposes of this paper are to (a) suggest a model for identifying critical thinking and metacognitive skills in evidence analysis instruction grounded in current educational theory and research and (b) demonstrate how freely available systematic review/meta-analysis tools can be used to focus on higher-order metacognitive skills, while providing a framework for addressing common student weaknesses. The final goal of this paper is to provide an instructional framework that can generate critique and elaboration while providing the conceptual basis and rationale for future research agendas on this topic.

## Background

Our goal in this article is to highlight an aspect of evidence analysis instruction that has been largely overlooked in the literature, namely, the role of metacognitive skills in the evidence analysis process. While research focuses on skills needed at the discrete steps in the evidence analysis process (e.g., searching, article assessment), skills needed to integrate the steps have gone largely unexamined. In this article, we present a conceptual framework we use to distinguish the different types of metacognitive skills within the evidence analysis instruction process and then provide two examples of how freely available online evidence analysis tools (Systematic Review Data Repository and OpenMeta[Analyst]) can be used to facilitate instruction in these metacognitive skills.

### Metacognition and self-regulation in the process of evidence analysis

Recent research in evidence analysis instruction focuses more on comparison of modes of delivery than on the relative benefits of different approaches to instruction grounded in alternative theories of adult learning or “andragogy” [1, 2]. This is not to say that courses for teaching evidence analysis skills ignore andragogical theory (e.g., see [3]), only that the ground of the instructional approaches is rarely made explicit. Lack of direction for more effective approaches for different audiences [1] may be due more to a lack of evidence [4] than concrete evidence that different approaches are more or less equivalent.

Our purpose here is to highlight one particular aspect of evidence analysis instruction which is, to the best of our knowledge, absent from the current literature: the role of metacognitive strategies in the integration of the commonly defined steps of the evidence analysis process. We provide a framework that locates both the place and the importance of these metacognitive skills in evidence analysis instruction and then provide an example of how free online tools can be leveraged to focus on these higher-order skills in graduate healthcare education. We ground our approach in adult learning (“andragogical”) theory [5, 6] emphasizing the role of both metacognition and self-regulation as meta-level elements of critical thinking [7, 8] involved in evidence analysis (though a detailed explication of these cognitive processes is beyond the scope of this article). Our hope is to provide a basis for critique, elaboration, and future research of this aspect of evidence analysis instruction. We offer our experience not as an exemplar or prescriptive, but as a worked, applied example that may provide a framework and rationale for further research in this area. For this report, no student level data were gathered and the Rutgers University IRB concluded that the regulatory definition of human subjects research was not met (ID 20140001119).

### Where do metacognitive skills fit in evidence analysis?

A key aspect for competence in the evidence analysis process is the critical integration of a set of skills toward a particular goal (whether the goal is production of a systematic review or meta-analysis (SRMA) or application to patient care [9]). Application of the evidence analysis process—whether in the production or consumption of evidence analysis products—specifically involves the integration of the skills of question formulation, evidence identification, analysis, critical synthesis and evaluation. Toward an integration, it is the higher-order cognitive skills, what we are calling *meta-strategic skills* [10], that regulate cognition (i.e., for planning, monitoring, and evaluation) and provide a mechanism for judgment and decision-making in the learning process [11–13]. Since successful application of evidence analysis skills requires students to master not just the discrete skills, but to *integrate* those skills toward a concrete goal, then self-regulated attention to these meta-strategic skills is needed.

**Fig. 1:**
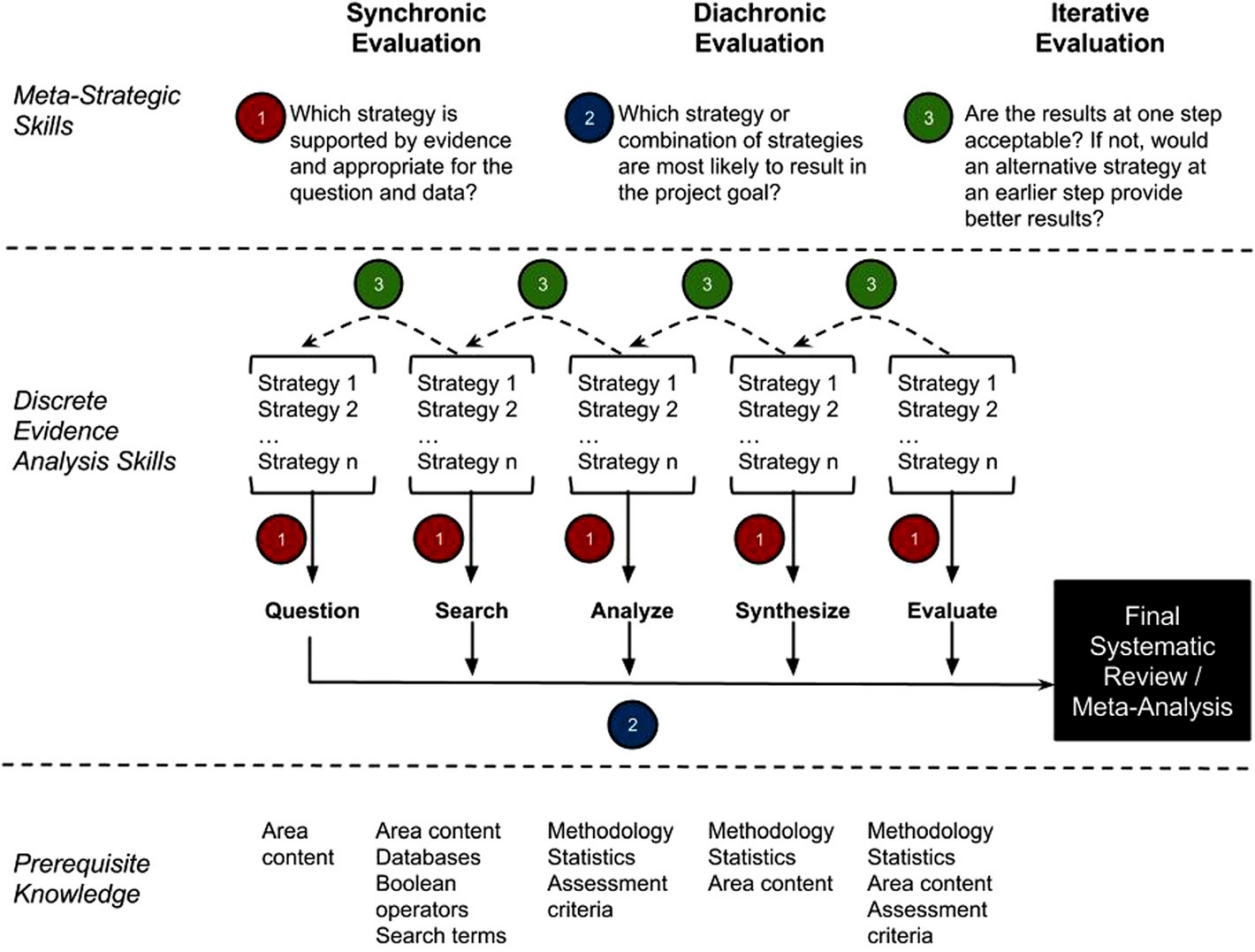
Conceptual framework of critical thinking in evidence analysis instruction

Research on cognitive skills, such as critical thinking, indicates that competence may be facilitated by making higher-order processes explicit within the context of instruction [14]. But what are these skills? How do they integrate into the process of evidence analysis instruction? In order to facilitate attention to these skills within the context of graduate healthcare education, we developed a conceptual framework (Fig. 1) that locates the types and roles of different meta-strategic skills within the evidence analysis process. We have found that students are more likely to understand and appreciate the quality of each evidence analysis step if they are required to link the product of a discrete step with earlier and subsequent steps, with the quality of each step’s product linked closely to upon the quality of that of surrounding steps. Feedback to students consists not only of the quality of a “stand-alone” product for a discrete step but also consists of feedback on *how well that product will link to subsequent steps* (e.g., not only how well a research question has been developed according to a PICOS framework, but also its relevance to healthcare goals, and its likelihood of having available relevant primary studies). Our goal is to have students internalize instructor feedback as a sort of self-talk where students regulate their own judgments of alternatives at each step as well as assess the results of each step in light of previous steps and planned goals [13].

We identify three general types of meta-strategic skills implicated in the evidence analysis process:

1. Synchronic evaluation: multiple tools, approaches, and platforms are available at each step of the evidence analysis process. Students should have the ability to identify and differentially evaluate which may be most appropriate for their purpose. For instance, which evaluation tool or which data extraction platform is going to be better for their purpose?
2. Diachronic evaluation: what is done at one step of the evidence analysis process (e.g., what data is extracted?) depends on the goals of later steps in the process (e.g., what type of analysis is planned?). Diachronic meta-strategic skills focus on teaching students how to look ahead to later steps to plan and execute activities at the current step of the process.
3. Iterative evaluation: unsatisfactory results at one step in the process (e.g., inadequate search results) may be a result of problems at an earlier step (e.g., poor question formulation). Students need to be able to assess the results of the evidence analysis at each step and evaluate how results in earlier steps may have led to current results.

We have found that using this framework helps to identify common problems in students’ evolving competence of the evidence analysis process toward production of a SRMA and provides a way to determine whether the problems are a result of inadequacy with a discrete evidence analysis skill or a result of a deficit in meta-strategic thinking (e.g., difficulty seeing how planned analyses shape information extracted from studies) [15].

Identifying explicit meta-strategic skills can be a first step toward recognizing common problems students face and, in our experience, assists in the mentoring process and in recommending additional student resources. What tools are available to facilitate the focus on these higher-order meta-strategic skills within the context of evidence analysis instruction and how can they be used to facilitate the development of meta-strategic skills? In the next section, we provide two examples of how a suite of evidence analysis tools can be used not simply to teach discrete evidence analysis skills but used to facilitate the development of potentially observable meta-strategic skills that we believe are integral for integrating these skills into a coherent process toward a concrete goal.

## Discussion

### Two brief examples: using evidence analysis tools to teach meta-strategic skills

There are many places within the instructional process where students can be encouraged to strategically reflect on their own thinking. Table 1 provides a schematic of how synchronic, diachronic, and iterative meta-strategic elements can be brought into play at the different steps of the evidence analysis process. In italicized text (Table 1), we provide examples of how tools available on the Systematic Review Data Repository (SRDR) platform can be used to facilitate these meta-strategic skills through a brief description of two of these tools—the SRDR for data extraction and management and OpenMeta[Analyst] (OMA)—within a multi-disciplinary, online, 15-week, health sciences graduate level evidence analysis course offered at Rutgers University, USA. To facilitate using these tools, students are assigned both a content advisor who is knowledgeable in their field of practice and a methodology advisor versed in evidence analysis and statistical methods. Our purpose is not to argue that this approach is how evidence analysis instruction should be offered, but rather to offer an example of how freely available evidence analysis tools can be leveraged to explicitly teach the meta-strategic skills we have argued are vital for learning evidence analysis. A key goal is to highlight where and how meta-strategic skills are involved so that these skills can be isolated, measured, and thus become the focus of future investigation in their own right [4].

**Table 1:** Examples of meta-strategic links and SRDR tools uses within the evidence analysis process

	Type of meta-strategic skill		
Step	Synchronic (strategies for weighing alternatives)	Diachronic (assessing current activities in light of downstream goals)	Iterative (evaluating current results based on previous activities)
Formulate question/conceptual framework (logic model)	Discriminate among question types	Relevance of question to practice	Identify need for preliminary background reading
	Discriminate among question components (PICO, PICOT, PIOS, etc.)	Availability of evidence to answer the question	
Identify evidence	Alternative data sources	Will available study designs answer the question?	Do too many or too few results indicate that the question was inadequately formulated?
	How will different methods of reporting outcomes affect the way the question can be answered?	Are search terms adequate to capture comparisons made at the analytic step?	If current SRMAs exist, how does the question for this SRMA provide new insight?
	*SRDR: Abstrakr facilitates consensus among project members for source selection.*		
Extract and analyze	Alternative platforms or extraction tools, basis for choosing among them	What design, sample, or intervention/exposure characteristics are necessary for later analyses or conclusions?	Do presence of common confounders suggests that the conceptual framework was misspecified?
	What methods of analyzing data are available? What are their relative benefits?	*SRDR: tabular structure scaffolds analytic framework for data extraction and a priori subgroup analyses.*	Do available outcome measures reported address the question asked?
	Are outcome measures commensurate?	*Outcome definition wizard motivates planning for type of analysis.*	*SRDR: Customizable questions allows for revision of logic model.*
	*SRDR: Customizable fields force planning at two levels: (1) information to be gathered and (2) structure of fields (multiple choice, numerical entry, free text)*		
Synthesize evidence	What methods of synthesis are available? What are their relative benefits and drawbacks?	How might the synthesis plan need to change in light of available data?	Does observed heterogeneity suggest that important extraction categories were missed?
	What are alternative methods of reporting outcomes?	Are sources of heterogeneity relevant for application identified?	*SRDR, OMA: high heterogeneity may indicate important moderator conditions missed in data extraction.*
	*SRDR: OMA wizard helps students identify appropriate method of meta-analysis.*	*SRDR: OMA facilitates post-hoc exploration of sources of heterogeneity.*	
Evaluate evidence	What are the various threats to confidence in the findings?		What aspects of analyses condition the application of findings?
			Were patterns between outcomes and study characteristics identified and analyzed?

Examples of use of SRDR suite of tools for evidence analysis instruction in italics

*PICO*, *PICOT*, *PIOS* question formulation heuristics comprising a combination of the following: population, intervention, comparator, outcome, time, or study design, *OMA* OpenMeta[Analyst], *SRDR* Systematic Review Data Repository, *SRMA* systematic review/meta-analysis

In the section below, we provide more details on two examples of how metacognitive skills can be integrated within the evidence analysis instruction process. We describe how the three meta-strategic skills (synchronic, diachronic, iterative) are implicated when formulating a conceptual framework and analyzing heterogeneity. As indicated in Table 1, metacognitive skills are implicated at other evidence analysis steps as well. So, the examples are meant to be illustrative, not comprehensive. Additionally, in order to increase the practical utility of our examples, we offer descriptions of our experience of the level of instructor involvement and the placement of the meta-strategic skill activities within the instruction sequence.

Our example uses two evidence analysis tools available from the Systematic Review Data Repository (SRDR) websites [16, 17]: the main website providing a free, customizable data extraction tool [18, 19] OpenMeta[Analyst], a free downloadable meta-analysis program [20]. While neither SRDR nor OpenMeta[Analyst] were deliberately designed as tools for teaching inexperienced students how to create SRMAs, both incorporate user supports (e.g., tutorials, wizards) relevant to operation of the applications, and both provide constructive scaffolding suitable not only for learning the process but suitable also for *teaching* more general evidence analysis and, crucially, meta-strategic skills.

### Example 1: developing a conceptual framework for data extraction

#### Synchronic skills

**Fig. 2:**
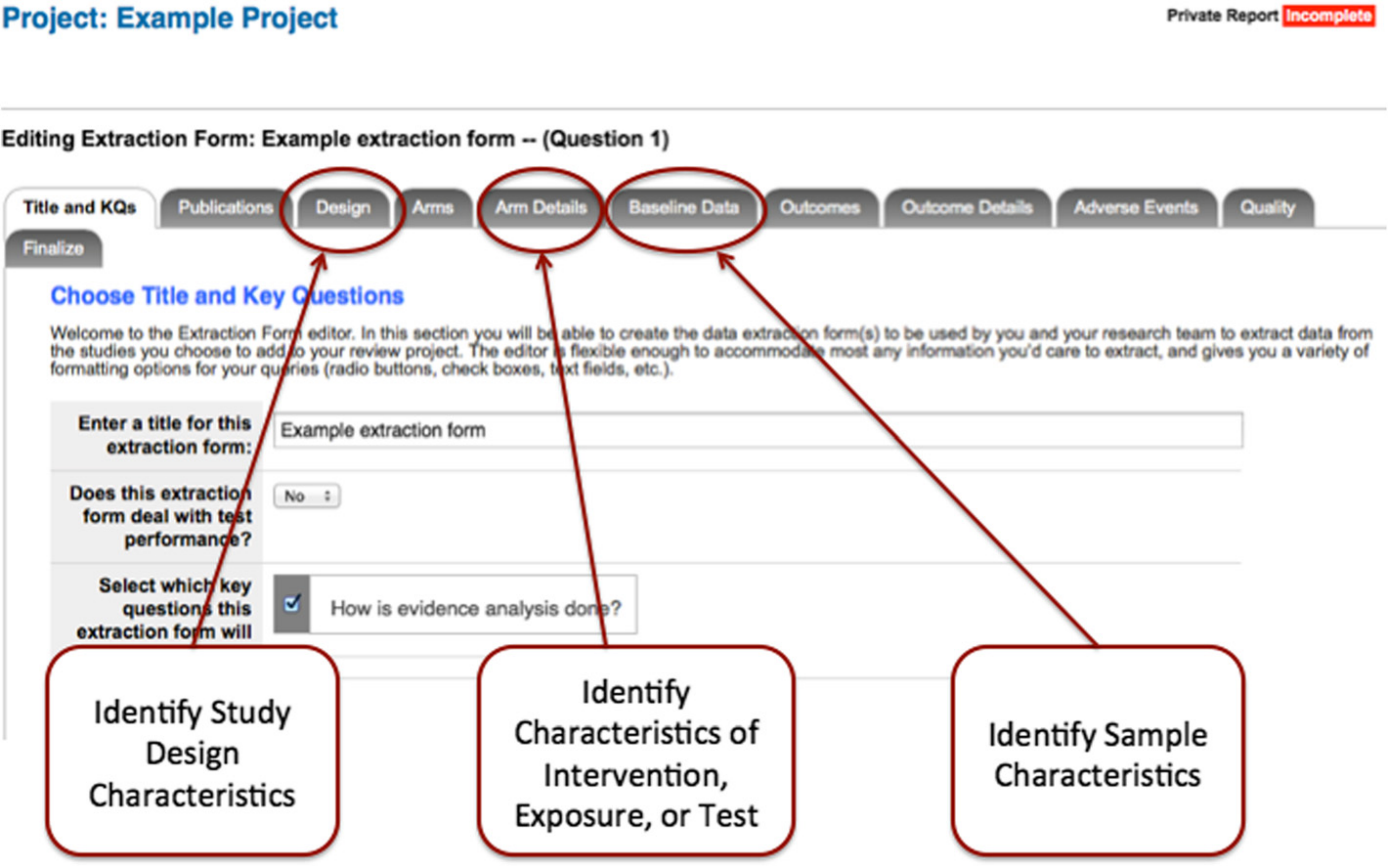
SRDR data extraction form creation screen

The act of creating a customized data extraction tool forces students to think strategically at two levels: what information do they need and what structure of data entry will be most useful and relevant. The SRDR tabular layout provides an a priori structured framework for the basic fields (Fig. 2). With the help of the content advisor and previous background reading on their topic, the student is pushed to consider “custom” fields relevant to their particular question and area of practice. Structured data entry options force students to consider types of responses. We have found that students left to their own devices tend to create a series of free text fields; while free text entry may sometimes be appropriate, data become messy when downloaded into a data matrix for analysis. Breaking fields up into smaller units can facilitate later analysis (e.g., rather than include a field called “treatment delivery characteristics”, a series of fields can be developed by students that codify duration, intensity (dosage), delivery personnel, common ancillary treatments, etc.).

#### Diachronic skills

Choosing among possible options for fields and field structure also has a temporal dimension. Beyond merely helping students recognize the data entry options available to them at a particular point, we encourage them to consider which of these alternatives should be included in light of their initial question and ultimate analytic goals. Students are encouraged to consider, during their initial reading, what aspects of study design, treatment/exposure characteristics, and sample characteristics could reasonably affect the outcomes. They are then encouraged to propose a small set of a priori subgroup analyses and determine what information would need to be systematically gathered from the studies in order to carry out those analyses. Collecting data on potential effect modifiers or context contingencies rarely occurs to the students without explicit instruction, but is facilitated by the scaffolding of the SRDR extraction tool.

#### Iterative skills

Customizable questions allow for revision of the conceptual framework or logic model. For example, as students read more deeply, they often intuit patterns they did not anticipate with the initial question (e.g., differences based on measurement device, based on subgroup, based on treatment or exposure characteristic). Potential confounders are identified that indicate that a more nuanced or detailed logic model is warranted. This highlights the problem with the ability to formulate an adequate question and logic model without substantial exposure to the content area. The initial questions are not always the best questions to be asking.

#### Instructor involvement

Instructor involvement at this stage of the process is intensive.

##### Methodological instructors

Providing refresher sessions on differences in study design and advising students on how the structure of fields will affect the form of downloaded data (and how this, in turn, will affect which analyses are feasible). Refresher instruction in differences in outcome measurement (categorical versus continuous versus time-to-event) and common statistical measures is often useful. Concrete examples of what different data structures look like in matrix format can help students see what they will be dealing with in coming sessions (e.g., show how data in spreadsheet form can be sorted on various characteristics and thus offer different perspectives on the outcomes). Feedback on student’s extraction template is facilitated by the collaborative features of the tool which enable ready access by instructors to each student’s SRDR extraction template, facilitating transparency of the student’s progress.

##### Content advisors

Specialists in the student’s field and content area are vital for helping students to understand and identify contingencies in treatment/exposure/diagnostic measurement that may affect the results of the study. Once the student has created an initial extraction framework, advisors provide detailed feedback using a rubric based on the structure of the SRDR tabular format. This allows the student to make targeted changes. Very few students create an adequate data extraction form on their first attempt.

#### Instruction sequence

We have found that it is helpful to first have students carry out a data extraction exercise on an article and extraction framework we provide. In this session, students focus on two skills: familiarizing themselves with the online platform (tabs, tools, resources) and study “interrogation” skills. This is followed by an exercise where students create their initial customized data extraction tool. At this step, the students are working on two key skills: (a) tying the creation of the data tool back to their initial logic model (i.e., the analytical framework) as well as forward to their planned analysis and (b) considering how to structure data entry to facilitate analyses. Because of the nature of diagnostic accuracy questions, and the substantially different methodological approach entailed, two separate “tracks” within the course offer tailored instruction for data extraction and framework construction for students asking diagnostic accuracy questions versus other types of question (e.g., treatment, prognosis, etc.).

### Example 2: understanding heterogeneity

#### Synchronic

**Fig. 3:**
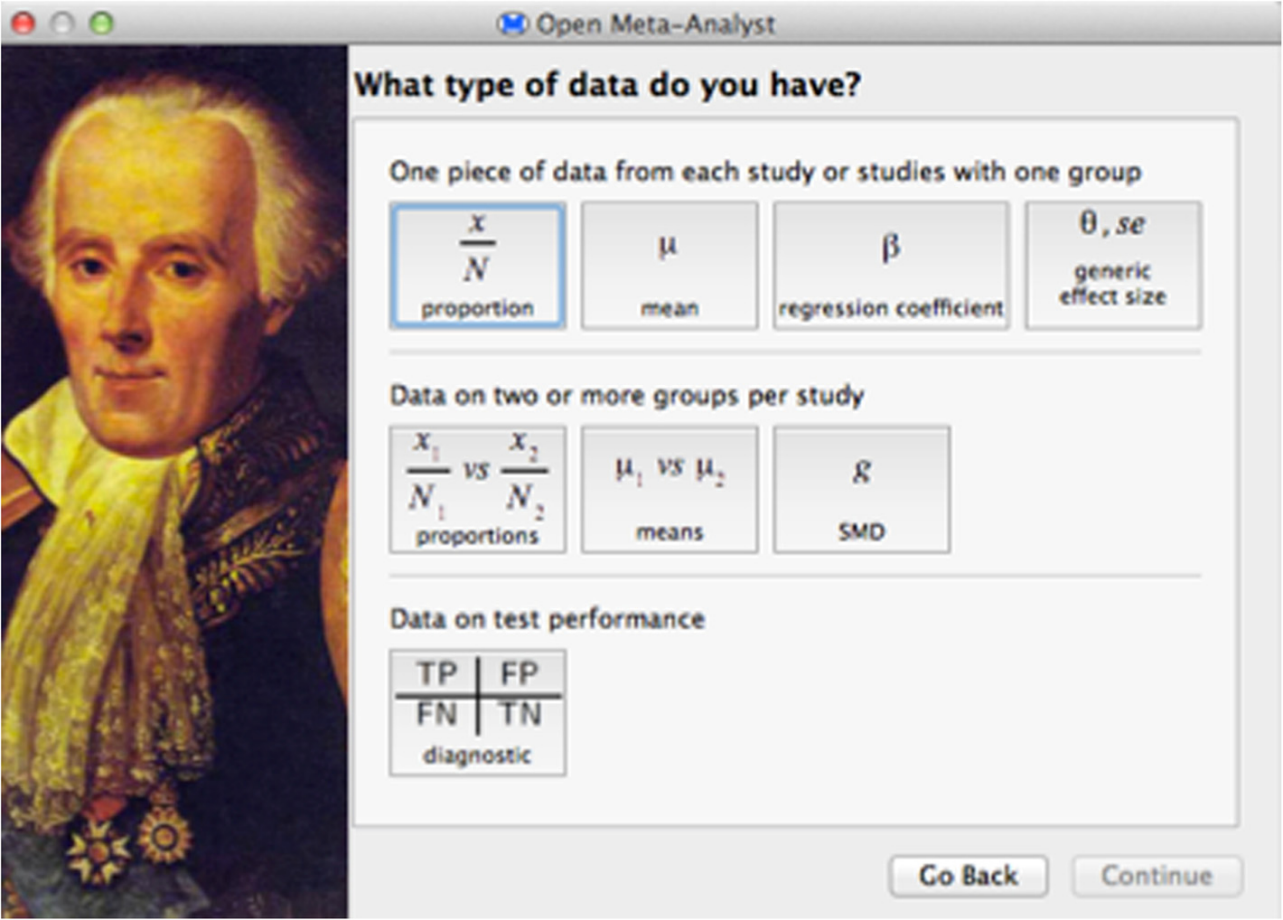
OpenMeta[Analyst] project setup wizard

Students are initially unaware of different approaches to meta-analysis and how the appropriate meta-analytic approach is shaped by the structure of the data. OMA provides a simple wizard (supplemented in class with a decision algorithm) to help students judge between alternative analyses based on the data they have collected (Fig. 3).

#### Diachronic

While students have been encouraged earlier to formulate a priori hypotheses regarding differences in results based on design, treatment, or sample characteristics, the results of their initial analyses often lead to post hoc exploration of other potential sources of heterogeneity as well as push them to consider how to explain differences among studies (e.g., one study may find very different results from the others) in the “Discussion” section of the paper. Students are encouraged to interpret their findings with respect to (a) suggesting explanations for studies that “do not fit,” (b) the level of heterogeneity found and implications for confidence, and (c) identifying explanations that make sense of the results in light of the relationships between individual study characteristics/findings and patterns across studies (IOM standard 4.2.4) and as their findings relate to the relevance of individual studies to the “populations, comparisons, cointerventions, settings, and outcomes or measures of interest” (IOM standard 4.2.5) [21].

#### Iterative

Students initially extract data in the format reported by authors. However, this format is not always commensurate with students’ specific research question and analytic goals. Thus, students are introduced to the notion that the measures needed for their meta-analysis can sometimes be computed or estimated from the information provided in the article to more closely fit their analytic needs with the use of a calculator available in OMA (or online calculators).

#### Instructor involvement

In our experience, instructor involvement outside course lectures and assignment grading is relatively light, as students become increasingly self-reliant with the content and mode of learning, and increasingly engage in conversations with their student colleagues in an interprofessional mix. Methodological instructors may be tapped for additional input on analysis or conversion of measures, especially when the student is drawing on observational studies. Because of statistical issues, students are discouraged from meta-regression, though this is permitted when the student has a stronger statistical background and is willing to do additional reading. Content advisors are occasionally asked for input regarding subgroup analyses.

#### Instruction sequence

Synthesis and interpretation is covered over the course of three sessions. The first introduces meta-analysis, and students complete an assignment with data provided to them (from a systematic review of treatment studies). The second session introduces them to subgroup meta-analysis and emphasizes the role of heterogeneity (and its analysis) in formulating more nuanced conclusions. Again, data are provided to the students, but this time on a diagnostic accuracy question where results are subgrouped by measurement device. This exposes students from all disciplines to meta-analytic procedures and statistical measures they might otherwise have little exposure to, as it has been observed in the course that presenting students with varying methodological examples reinforces learning in a constructivist sense.

## Conclusions

We offer the above conceptual framework and examples of meta-strategic instruction in action as a point of departure for critique, elaboration, and future research. Indeed, if we want to be able to empirically test whether an explicit focus on meta-strategic skills in evidence analysis instruction leads to better mastery of the discrete evidence analysis skills or a more nuanced approach to appraising the quality of systematic reviews and meta-analyses, then we must first have some sense of what these skills are and how they may come into play. In short, before we can test, we must first identify and measure the metacognitive processes involved in evidence analysis.

In our experience, encouraging students to attend critically to their thinking about the *integrative* process of evidence analysis has resulted in higher-quality performance, both in terms of discrete skills as well as the final product (a limited scope SRMA). We recognize, however, that our approach may not fit every situation, and several questions remain to be explored: Whether or to what degree can a focus on metacognitive skills be applied in other instructional settings? What student outcomes result from this meta-strategic focus? For what kinds of adult learners is this focus appropriate?

## Abbreviations

OMA: OpenMeta[Analyst]
PICO: question formulation heuristic comprising: population, intervention, comparator, outcome
PICOT: question formulation heuristic comprising: Population, intervention, comparator, outcome, time (duration of data collection)
PIOS: question formulation heuristic comprising: population, intervention, outcome, study design
SRDR: Systematic Review Data Repository
SRMA: systematic review/meta-analysis
